# Removal of brilliant green dye from synthetic wastewater under batch mode using chemically activated date pit carbon

**DOI:** 10.1039/d0ra08488c

**Published:** 2021-02-17

**Authors:** Ramadan Abd El-Ghany Mansour, Mohamed Gamal Simeda, Ahmed Amin Zaatout

**Affiliations:** Basic Sciences and Engineering Department, Higher Institute of Engineering and Technology New Damietta Egypt drramadanelkatep@yahoo.com; Chemical Engineering Department, Higher Institute of Engineering and Technology New Damietta Egypt eng.modysmsm@gmail.com; Chemical Engineering Department, Faculty of Engineering, Alexandria University Egypt draazaatout@yahoo.com

## Abstract

In this research, a single-stage batch adsorber was designed for removal of brilliant green dye (BG) from aqueous solutions using activated carbon derived from date pits (ADPC) based on the Freundlich isotherm which was the best-fitted isotherm model. Experimental work was carried out within the range of 10–50 ppm initial dye concentration to determine the optimum operating conditions which were 55 min contact time, 0.06 g adsorbent mass, 25 °C, and pH = 8. Process kinetics was best-fitted with the pseudo-second order model, which revealed that the intra-particle diffusion stage is the rate-controlling stage for the process. The process efficiency was assessed by infrared spectroscopy (FTIR), scanning microscopy (SEM), X-ray spectroscopy (EDXS), and Brunauer–Emmett–Teller (BET) where the latter showed that the specific surface area of the adsorbent is 311.38 m^2^ g^−1^, which gives a favorable maximum monolayer adsorption capacity (77.8 mg g^−1^). The thermodynamic study proved that BG adsorption on ADPC was physiosorptive (Δ*G* = −5.86 kJ mol^−1^) and spontaneous at low temperature (Δ*H* = −17.7 kJ mol^−1^, Δ*S* = −0.04 kJ mol^−1^ K^−1^).

## Abstract 2

In this research, a single-stage batch adsorber was designed for removal of brilliant green dye from aqueous solutions using activated carbon derived from date pits based on the Freundlich isotherm which was the best-fitted isotherm model.

## Graphical abstract

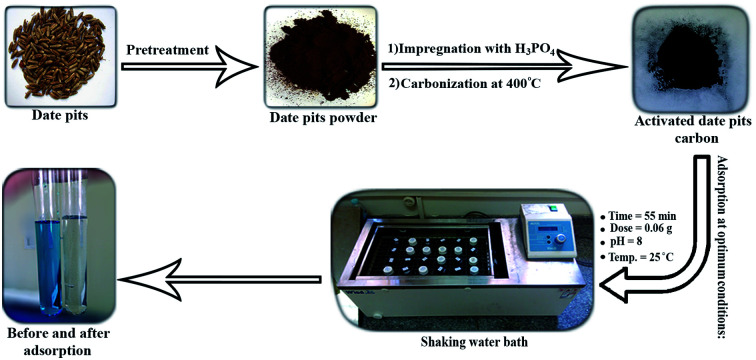

## Introduction

Coloring agents and dyestuffs have become the main feedstock for the production of domestic goods, colorful fabrics, paints, printing inks, and so on.^1,2^ For instance, the total textile dye consumption is greater than 10^7^ kg per year worldwide with roughly 10^6^ kg per year of dyes discharged into water streams killing more than three million people, mainly infants, who get water for drinking and irrigation.^3,4^ Several kinds of industrial dyestuffs can be found in wastewater and it is classifiable into three major groups: anionic (direct, acid, and reactive dyes), cationic (basic dyes) and non-ionic (disperse and vat dyes)^5^ while in general, cationic dyes have the most harmful and toxic effects on the ecosystem of receiving water and the whole environment.^6^ Releasing such toxic dyes into the environment has become a worldwide concern because of their chemical stability,^7^ and cancer-causing and mutagenic impacts that influence oceanic biota by preventing light from penetrating through water, which slows down photosynthetic activity and also tends to toxify fish and other organisms due to chelating metal ions. Oxidation and reduction of these dyes in water can also produce hazardous substances that raise the necessity of getting rid of them from wastewater.^8,9^ They also cause hyperactivity, low frustration tolerance, impulsivity, and lack of attention when they are used as food additives.^10^ For that reason, numerous methods such as chemical oxidation, electrolysis, coagulation, ozonation, reverse osmosis, ultra-chemical filtration, ion exchange, precipitation, flocculation, biological methods, and many others have been used to eliminate organic pollutants.^11,12^ However, their use on a large-scale is restricted due to their long operational time, high production cost, and toxic side products.^13,14^ Adsorption has been observed to be better than multiple techniques for treating wastewater contaminated with dissolved substances and drugs^15,16^ because it is inexpensive, very flexible, modest in design, easy to operate, and capable of handling relatively high flow rates of water without production of residual sludge or pollutants.^17,18^ Moreover, successful water pollutant adsorption provides their recovery, which is especially required for water nutrients.^19^ Adsorption techniques can be classified into batch which is a simple technique that offers a convenient way to better explain the parameters that control the adsorption mechanism or continuous technique which is useful in evaluating the amount of adsorbent required to extract the wastewater contaminants.^20^ Commercial activated carbon is a commonly utilized adsorbent in waste-water treatment processes as it consists of a micro-porous homogenous structure that gives large surface area, high adsorption capacity, and high porosity^21^ but its wide utilization was restricted because it is costly and its uptake capacity and yield after regeneration decreased by 10–15%.^22^ Hence, scientific researchers have focused their attention on producing activated carbon from renewable domestic agricultural waste because of its low preparation costs.^23,24^ “Table 1” shows the advantages and disadvantages of previously reported biomass utilized in wastewater treatment compared with date pits. Physical and chemical activation are the basic processes for producing activated carbon. The first technique is composed of raw material carbonization and gasification by steam or carbon dioxide of the resulting char. The second technique consists of impregnation of the precursor material with chemical reagent (KOH, ZnCl_2_, H_2_SO_4_, H_3_PO_4_, HNO_3_, and CO_2_) then carbonization of the impregnated product.^32^ ZnCl_2_ and H_3_PO_4_ appear to be the most used reagents for activated carbon preparation but H_3_PO_4_ is preferred because of its ease removing by washing with warm or cold water after carbon activation.^33^ Moreover, the iodine number of carbon prepared from H_3_PO_4_ is greater than the iodine number of carbon prepared from ZnCl_2_.^34^ The chemical activation process is superior to the physical activation process because of its high productivity and high surface area of the resulted carbon^9^ in addition to its low energy costs.^35^ BG is an odorless cationic dye in the form of golden-green crystals.^4^ It is known as Diamond green G, Solid green, Ethyl green, and Basic green 1. It can be used as a biological mark, dermatologic agent, antimicrobial treatment, and inhibition of mold propagation in poultry food.^36^ It is also widely utilized in the dyeing of textiles and manufacturing of inks for printing papers. Approximately 0.8–1.0 kg of BG is utilized per ton of produced paper sheet.^37^ For mankind, BG causes gastrointestinal tract discomfort which leads to nausea, vomiting, and diarrhea. It also induces cough and shortness of breath in the respiratory tract. Moreover, it can cause skin irritation with redness and ache in case of direct contact.^38^ Decomposition of BG may produce hazardous gases like CO, CO_2_, NO, NO_2_, and SO_2_.^5^ As a result, a lot of researchers studied the adsorption of BG from wastewater by carbonized and non-carbonized as given in “Table 2”. Date pits can be considered a waste product after processing of compressed dates (fresh date fruit has 10–15% seed from its weight).^39^ It was used as carbonized and non-carbonized compound for many adsorption kinds of research to remove industrial dyes such as Methylene Blue,^40^ Indigo Carmine,^9^ Methyl Orange,^35^ Disperse blue dye,^41^ Maxilon blue,^23^ and Congo red.^42^ The current survey was aimed to determine the optimum contact time, adsorbent mass, temperature, pH, and initial BG concentration. Some adsorption isotherm models were studied to know the maximum adsorption capacity for the activated date pits carbon and the best-fitted model for the process. The kinetic and thermodynamic studies were investigated to define the mechanism and the rate-controlling stage for the process. The experimental data was well fitted with Freundlich isotherm which was used in designing a batch adsorber with one stage.

**Table 1:** Comparative study between previously reported biomass utilized in wastewater treatment and date pits

Adsorbent	Advantages	Disadvantages
Wood^25^	Produced activated carbon has a highly porous structure (*S*_BET_ = 1884 m^2^ g^−1^) and useful functional groups	The yield of produced activated carbon is low
	Produced activated carbon can make a rapid adsorption process with high adsorption capacity	Wood can be more useful in production of furniture
Bagasse^26^	Produced activated carbon is very efficient for the adsorption of gases due to its excellent stability and regenerability	Produced activated carbon can't be useful in dyes or heavy metals removal because of its non-sufficient functional groups
	Produced activated carbon has a large specific surface area (*S*_BET_ = 1113 m^2^ g^−1^)	Produced activated carbon has a small micro-pore diameter (less than 1 nm)
Orange peel^27^	Produced activated carbon gives high adsorption capacities.	The yield of produced activated carbon is low
	Produced activated carbon has many useful functional groups	Produced activated carbon requires several treatment processes to control the ash content
Biological sludge^28^	Produced activated carbon has a large specific area and high micro-pore diameter	The yield of produced activated carbon is low due to high liquid content
	Produced activated carbon gives high adsorption capacities	Produced activated carbon can't be useful in heavy metals removal because of its non-sufficient functional groups
Rice husks^29^	Pyrolyzed rice husk can be used to remove color and turbidity of wastewater	Produced activated carbon has a small specific surface area and small adsorption capacity
	Raw rice husk has been reported to remove 98.24% of humic acid from aqueous solutions	Produced activated carbon requires several pre-treatment processes to control the ash content
Waste palm shells^30^	The yield of produced activated carbon is very high (97.8 wt%) and the activation time is very small (10 min)	Produced activated carbon has a small specific surface area and small adsorption capacity
	Produced activated carbon doesn't need several treatment processes due to low ash content	Produced activated carbon has non-sufficient functional groups
Coconut husk^31^	Produced activated carbon has a large specific surface area (*S*_BET_ = 1448 m^2^ g^−1^)	Raw coconut husk can be more useful when used as a fuel for household purposes
	Produced activated carbon can be regenerated without any damage (desorption efficiency >95%)	Raw coconut husk needs several purification processes to eradicate dirt and other contaminants
Date pits (present work)	Produced activated carbon has a convenient specific surface area and adsorption capacity	Grinding of raw date pits need a special mill for hard rocks
	The yield of produced activated carbon is high (>70 wt%)	
	The purification processes of raw date pits are very simple.	
	Produced activated carbon can be useful in dyes or heavy metals removal due to its sufficient functional groups	

**Table 2:** Reported adsorption capacities (*Q*_m_) of BG by different adsorbents

Adsorbent	Maximum adsorption capacity (mg g^−1^)
Red clay^6^	125
Bambusa Tulda^43^	41.67
Sandpaper wastes^44^	294.1
Radish peels^5^	0.069
Coal^5^	0.929
Montmorillonite^45^	229
Cellulose^46^	150
Chemically activated guava seeds carbon^47^	80.45
Chemically activated date pits carbon (present work)	77.76

## Materials and methods

### Activated date pits carbon preparation

The raw date pits were collected from a factory that produces compressed dates (Agwa) in Damietta, Egypt. The boiled water was used in washing the pits to remove any date residue. The pits were dried at 110 °C in an electric oven to make them free from moisture then ground to a fine powder using (RETSCH lab mill). The raw date pits powder was placed in a glass container and a concentrated phosphoric acid (85%) was poured carefully into the container until 20 g of seeds will be impregnated in 40 mL of H_3_PO_4_ at 25 °C for 24 hours. At the end of the soaking time, the soaked seeds were dried at 110 °C for one hour. The seeds were placed on a ceramic cup and subjected to 400 °C for 2 hours in a muffle furnace (Hobersal JB-20). Then, neutralization of the seeds was carried out using hot distilled water and after that, the produced activated carbon was dried at 110 °C for 3 hours to remove any undesired moisture within the particles. Finally, the activated date pits carbon was sieved using (SAMA Sieve Shaker) to obtain an equal particle diameter which was (75 μm).

### Preparation of the adsorbate

BG (C_27_H_34_N_2_O_4_S) was bought with molecular weight 482.62 and was used by dissolving 1 g of BG powder, weighed by a four-digit analytical balance (KERN-ABS 220-4, UK), in 1 L of distilled water to prepare the stock solution (1000 ppm). Diluting the stock solution with distilled water was carried out to prepare the desired experimental concentrations. The wavelength was measured to be 625 nm and the final concentrations were measured using (PG instrument Ltd T80, UK) UV-Visible spectrophotometer.

### Experimental studies

**Fig. 1:**
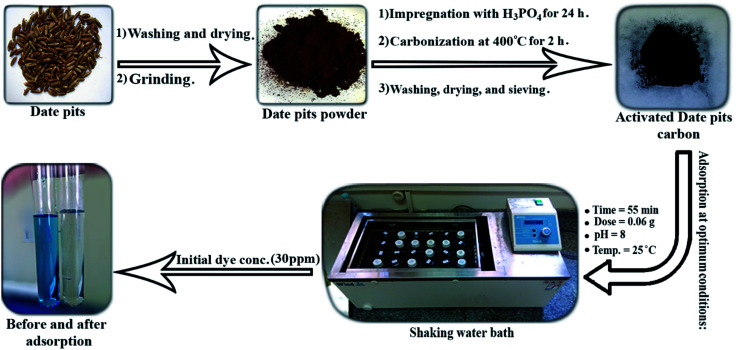
Graphical abstract for the process.

The batch adsorption process was carried out to study the effect of the experimental parameters (contact time, adsorbent mass, temperature, pH, and initial BG concentration) on the adsorption of BG by activated date pits carbon (ADPC) and can be summarized in “Fig. 1”.

Firstly, a 0.02 g of ADPC was contacted with 50 mL of a BG solution at different concentrations (10–50 ppm) at neutral pH in 125 mL glass bottles at a fixed temperature (25 ± 2 °C) and constant shaking speed (240 rpm) in a shaking water bath (Wisd laboratory instruments, DAHAN Scientific co., Ltd, 30, Korea) for different intervals of time until it reached the equilibrium at (55 min). Secondly, the solutions were centrifuged using (80-1 Electric Centrifuge) and the concentrations of the filtrated samples were determined by the spectrophotometer. The previous procedures were applied at (0.01–0.08 g) of ADPC and constant time (55 min) until the equilibrium was observed at (0.06 g) which is the optimum adsorbent mass. The temperature effect was studied by applying the above procedures at 55 min contact time, 0.06 g dose, neutral pH, and different temperatures (25–95 °C). The initial concentration effect was determined by preparing (10–100 ppm) from the stock of the dye solution and applying the repeated process at the optimized conditions of the previous parameters (55 min time, 0.06 g dose). The pH parameter was investigated by adjusting the pH of the dye solutions using a pH-meter (Hanna-Instruments 8519, pH 211, Canada), HCl, and NaOH and repeating the process as described above at equilibrium time and dose until it gives the highest removal percent which was at pH = 8. The process efficiency was determined using the following equations:

1 (% *R*) = [1 − (*C*_e_/*C*_o_)] × 100

2 *Q*_e_ = (*C*_o_ − *C*_e_)*V*/*W*

where “*C*_o_” is the initial concentration of BG solution in (ppm), “*C*_e_” is the final concentration of BG solution in (ppm), “*V*” is the volume of BG solution in (L), and “*W*” is the mass of the adsorbent in (g). “*Q*_e_” is the adsorption capacity at equilibrium (mg g^−1^).^47^

## Results and discussion

### Characterization of activated date pits carbon

**Fig. 2:**
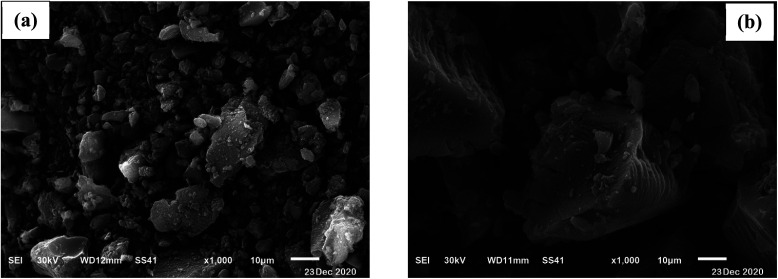
Scanning Electron Microscope of activated date pits carbon (ADPC) (a) before adsorption and (b) after adsorption of BG.

SEM analysis was used to obtain surface morphology of activated date pits carbon (ADPC) before and after adsorption of BG by (JEOL JSM 6510 lv, Japan). “Fig. 2a” displays a heterogeneous morphology of ADPC before adsorption of BG including many small pores that are distributed randomly on the surface. “Fig. 2b” clearly shows the aggregation of BG particles on the surface of ADPC which makes the pores mostly disappeared and this indicates that BG was successfully adsorbed on the surface of ADPC.^47^

EDXS analysis was used to clarify the qualitative elemental composition of activated date pits carbon (ADPC) before and after adsorption of BG by (JEOL JSM 6510 lv, Japan). According to the X-ray spectroscopy, the chemical composition (in wt%) of ADPC unloaded with BG was 76.73 for carbon, 21.92 for oxygen, 0.19 for sodium, 0.56 for phosphorus, 0.39 for copper, and 0.22 for zinc. Furthermore, the chemical composition (in wt%) of ADPC loaded with BG was 79.38 for carbon, 19.06 for oxygen, zero for sodium, 0.82 for phosphorus, 0.42 for copper, and 0.33 for zinc. Based on the noticeable differences in weight percents between ADPC loaded and unloaded with BG, it can be concluded that the chemical structure of ADPC was changed due to the effective adsorption of BG molecules on the pores of ADPC.^47^

FTIR analysis was used to see how far the molecular structure of activated date pits carbon (ADPC) has been changed after adsorption of BG by (ThermoFisher Nicolete IS10, USA). According to the IR spectrum of ADPC before adsorption of BG, there are some strong, moderately strong, and weak bands at 3407, 2920, 2851, 1602, 1435, 1376, 1230, 1077, 507, and 421 cm^−1^ assigned to O–H stretch, methylene C–H asymmetric stretch, methylene C–H symmetric stretch, primary amine N–H bend, methyl C–H asymmetric bend, methyl C–H symmetric bend, aromatic ethers aryl-O stretch, C–O–C cyclic ethers, aryl disulfides S–S stretch, and aryl disulfides S–S stretch. On the other hand, the IR spectrum of ADPC after adsorption of BG has strong, moderate, and weak bands at 3431, 2922, 2853, 1617, 1579, 1474, 1411, 1380, 1337, 1251, 1214, 1181, 1152, 1070, and 894 cm^−1^ assigned to O–H stretch, methylene C–H asymmetric stretch, methylene C–H symmetric stretch, primary amine N–H bend, C=C–C aromatic ring stretch, C=C–C aromatic ring stretch, vinyl C–H in-plane bend, methyl C–H symmetric bend, methyne C–H bend, aromatic ethers aryl-O stretch, P–O–C aromatic phosphates stretch, R-O-SO_3_ organic sulfates, R-SO_3_ sulfonates, C–O–C cyclic ethers, C–H vinylidene out-of-plane bend.^48–51^ The observed shifts of the bands in the IR spectrum to higher and lower wavelength as well as the appearance of new bands at 1579, 1474, 1337, 1214, 1181, 1152, and 894 cm^−1^ and disappearance of some bands at 507, and 421 cm^−1^ can be considered strong evidence for adsorption of BG on the surface of ADPC.^47^

### Operating conditions effect

**Fig. 3:**
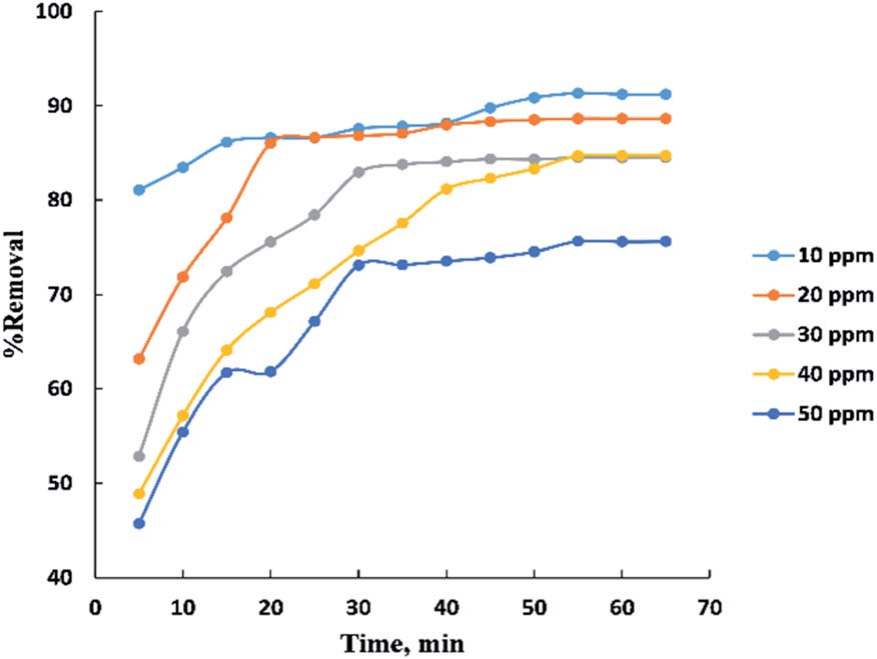
Effect of contact time on the adsorption of BG dye onto ADPC.

Process time can be represented by “Fig. 3” for adsorption of different concentrations (10–50 ppm) of BG onto a fixed weight (0.02 g) of activated date pits carbon (ADPC) in the range of (5–65 min). It can be observed that the low concentrations (10 and 20 ppm) reach the equilibrium very rapidly at the first twenty minutes while the high concentrations (30, 40, and 50 ppm) reach the equilibrium at (55 min) after a gradual increase in the removal percent during the time shown in the figure and this could be interpreted by the theory that in adsorption of dyes, firstly, the dye molecules have to compete against the effect of boundary layer interface. Secondly, the penetrating dye molecules move out from the boundary layer film to distribute onto the surface of the adsorbent. Finally, the remaining dye molecules diffuse into the porous structure of the adsorbent. Based on this theory we conclude that when the initial concentration of dye solutions increases, the number of dye molecules increases, so the process will take a relatively long contact time to reach the equilibrium state.^6^

**Fig. 4:**
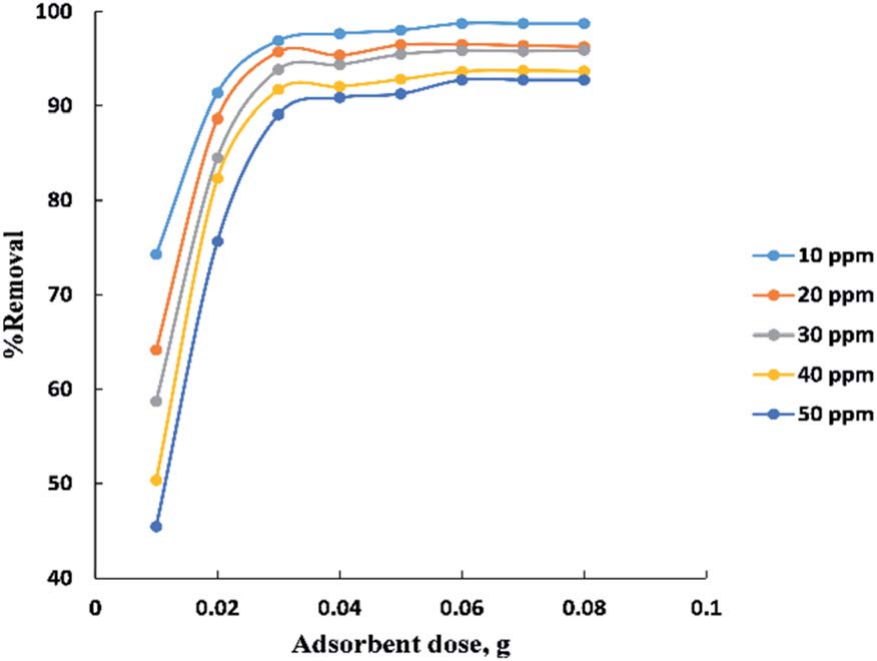
Effect of adsorbent dose on the adsorption of BG dye onto ADPC.

The adsorbent dose parameter was displayed in “Fig. 4”, the removal percent of (10–50 ppm) BG onto activated date pits carbon (ADPC) increases with the increase in adsorbent dose ranged from (0.01–0.06 g) due to the availability of more adsorbent active sites as well as greater availability of specific surface area of the adsorbent. After that, there are no significant changes in removal percent were observed due to aggregation of adsorption sites takes place which makes the total ADPC surface area decrease, leading to a slighter increase in BG removal.^52^ So, (0.06 g) can be considered the optimal dose for ADPC loading.

**Fig. 5:**
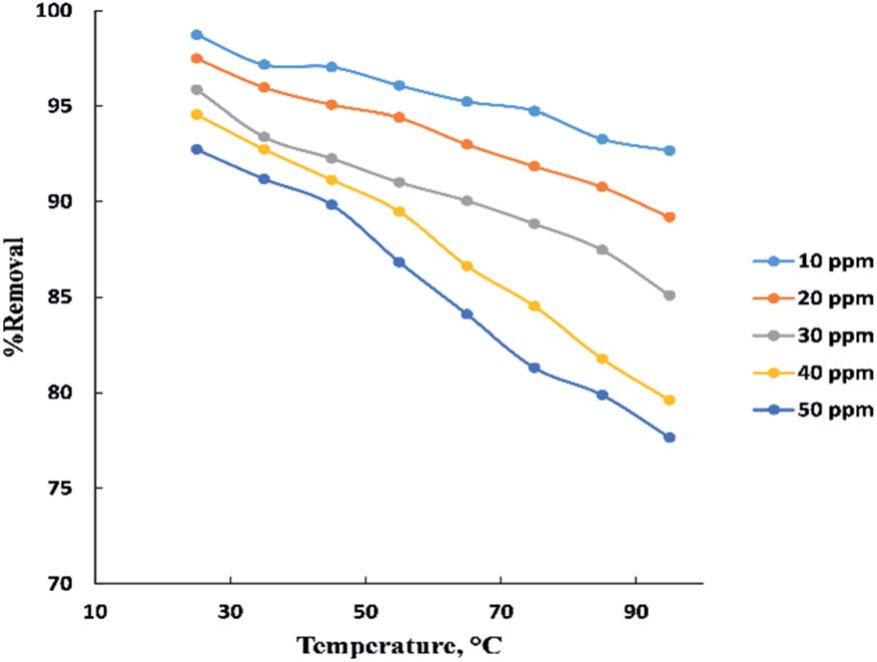
Effect of temperature on the adsorption of BG dye onto ADPC.

The temperature parameter is commonly used to indicate if the process is endothermic or exothermic. “Fig. 5” displays the temperature influence in neutral medium (pH = 6–7) for adsorption of BG at (10–50 ppm) on the surface of activated date pits carbon (ADPC) with constant weight (0.06 g). It is clearly shown that as temperature increased in the range (25–95 °C), the removal percent of BG decreased from 98% to 77%. This may be due to weakness of physical bonding between the dye molecules and the active sites of the adsorbent with increasing temperature. Also, as temperature increases, the solubility of dye increases, the interaction forces between the solute and the solvent become stronger than solute and adsorbent, so the solute is more difficult to adsorb. Therefore, it can be concluded that the process is exothermic.^8,40^

**Fig. 6:**
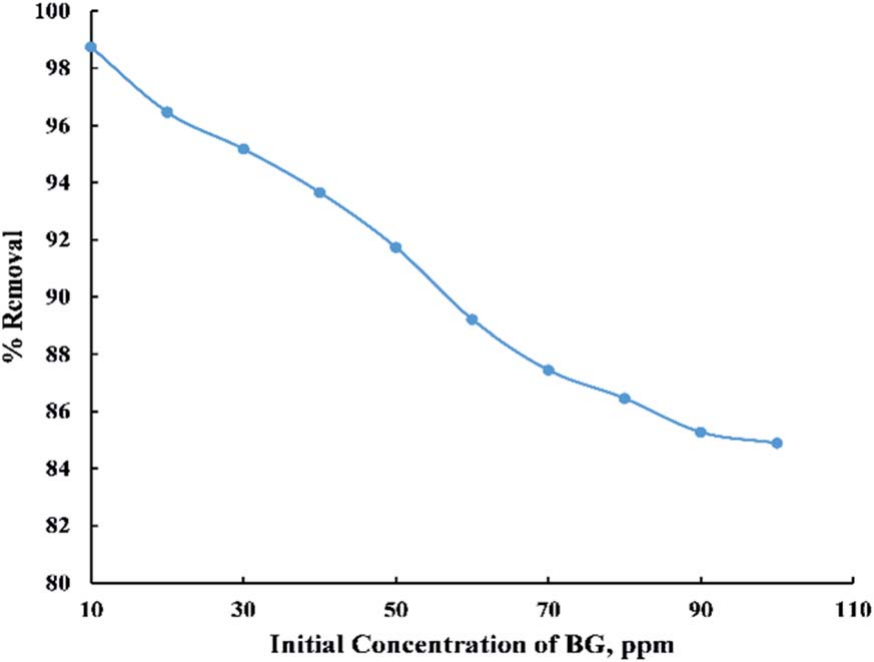
Effect of initial dye concentration on the adsorption of BG dye onto ADPC.

The initial BG concentration parameter was studied within the range (10–100 ppm) at 25 °C, 0.06 g adsorbent dose, and 55 min contact time. From “Fig. 6” it was observed that the removal percent decreased sharply with the increase in initial concentration up to 90 ppm then it noticed that there were no considerable changes in the removal percent and this can be explained by the fact that at low initial dye concentration the number of active sites on the adsorbent's surface is more available compared with high initial dye concentration. So, most of the dye molecules have been adsorbed on the surface of activated date pits carbon (ADPC) leading to higher percentage removal while at high initial BG concentration, molecules have low chances to be adsorbed due to the limited number of binding sites at the adsorbent's surface. Thus, some of the dye molecules remain in the solution and do not get adsorbed.^6^

**Fig. 7:**
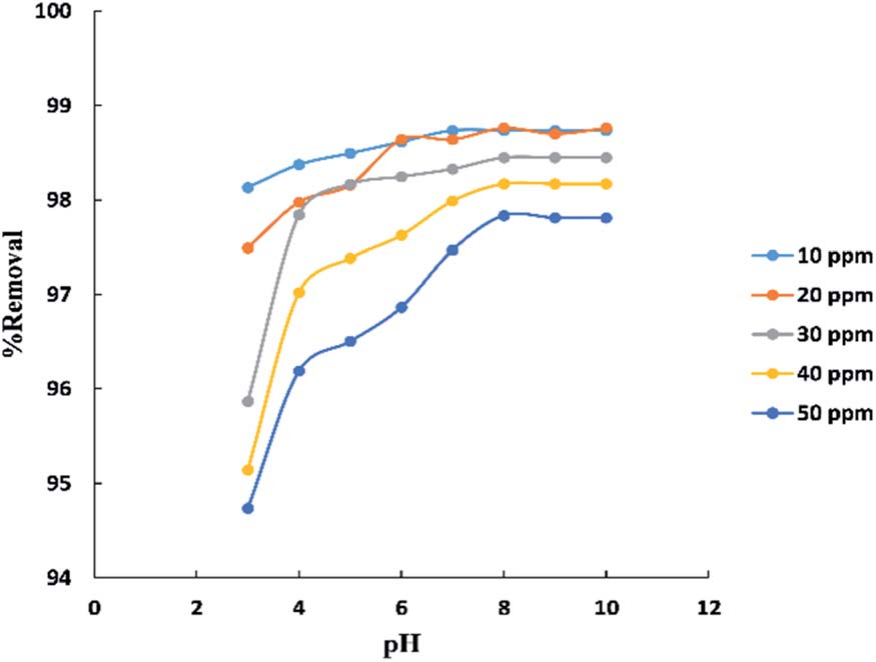
Effect of pH on the adsorption of BG dye onto ADPC.

The pH parameter was investigated within the range (3–10) at (10–50 ppm) initial concentration of BG and room temperature (25–30 °C) as shown in “Fig. 7”. The results indicate that the removal percent of BG increases with the increase in pH up to pH 8 after which was observed no significant improvement in the process. This may be explained that at lower pH the surface charge of activated date pits carbon (ADPC) may get positively charged which make H^+^ ions compete effectively with dye cation leading to a decrease in the removal percent of dye adsorbed while at higher pH, the surface of ADPC may get negatively charged which enhances the electrostatic force of attraction between the positively charged dye cation and the adsorbent's surface.^53^ Also, at higher or lower pH, as the initial dye concentration increases, the competition between dye molecules to be adsorbed increases, so the removal percent decreases.^40^

### Isotherm and equilibrium study

The isotherm study was carried out by fitting the experimental data to four isotherm models and find a convenient model that can be used for design purposes using the correlation coefficients, *R*^2^ values as shown in “Table 3”.

**Table 3:** Adsorption isotherm of BG dye onto activated date pits carbon (ADPC) at 298 K

Isotherm models	Parameter value	Isotherm models	Parameter value
Langmuir model		Freundlich model	
*Q*_m_ (mg g^−1^)	77.75539	*K*_f_ (mg g^−1^)	20.0302
*K*_L_ (L mg^−1^)	0.306076	*n* (g L^−1^)	2.240371
*R*_L_	0.246259	1/*n*	0.446355
*R* ^2^	0.9492	*R* ^2^	0.9973
Temkin model		Dubinin–Radushkevich model	
*K*_t_ (L mg^−1^)	6.873598	*K*_t_ (L mg^−1^)	6.873598
*q*_m_(J mol^−1^)	12.95402	*q*_m_ (J mol^−1^)	12.95402
*R* ^2^	0.9005	*R* ^2^	0.9005

**Fig. 8:**
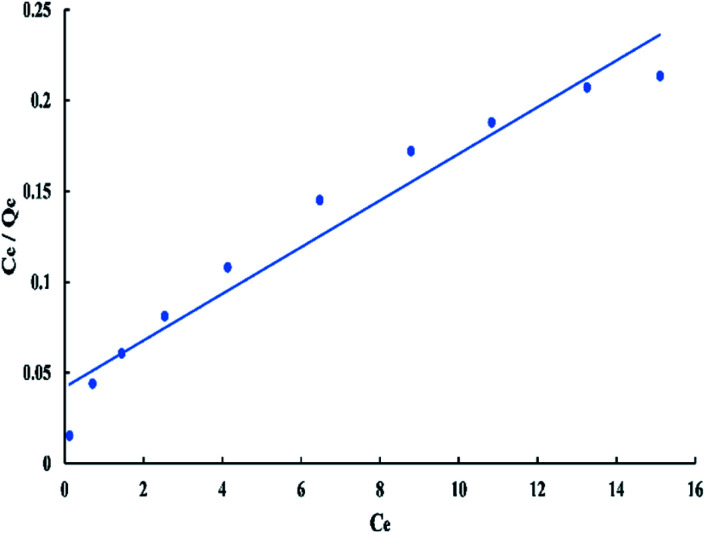
Langmuir isotherm for adsorption of BG onto ADPC at 298 K.

For Langmuir model, *C*_e_/*Q*_e_*versus C*_e_ has a linear relationship as shown in “Fig. 8”, with a slope equal to 1/*Q*_m_ and an intercept equal to 1/(*Q*_m_*K*_L_). The linear Langmuir equation is given as:

3 *C*_e_/*Q*_e_ = 1/(*Q*_m_*K*_L_) + *C*_e_/*Q*_m_

where “*Q*_m_” is the maximum adsorption capacity (mg g^−1^), and “*K*_L_” is the Langmuir affinity constant (L mg^−1^) related to “*Q*_m_” and rate of adsorption. The feasibility of the process can be evaluated by a separation factor (dimensionless constant) “*R*_L_” which is given in the following equation:

4 *R*_L_ = 1/(1 + (*K*_L_*C*_o_))

The “*R*_L_” value lays between zero and one for favorable adsorption, whereas (*R*_L_ > 1), (*R*_L_ = 1), and (*R*_L_ = zero) for unfavourable, linear, and irreversible adsorption respectively.^40^

**Fig. 9:**
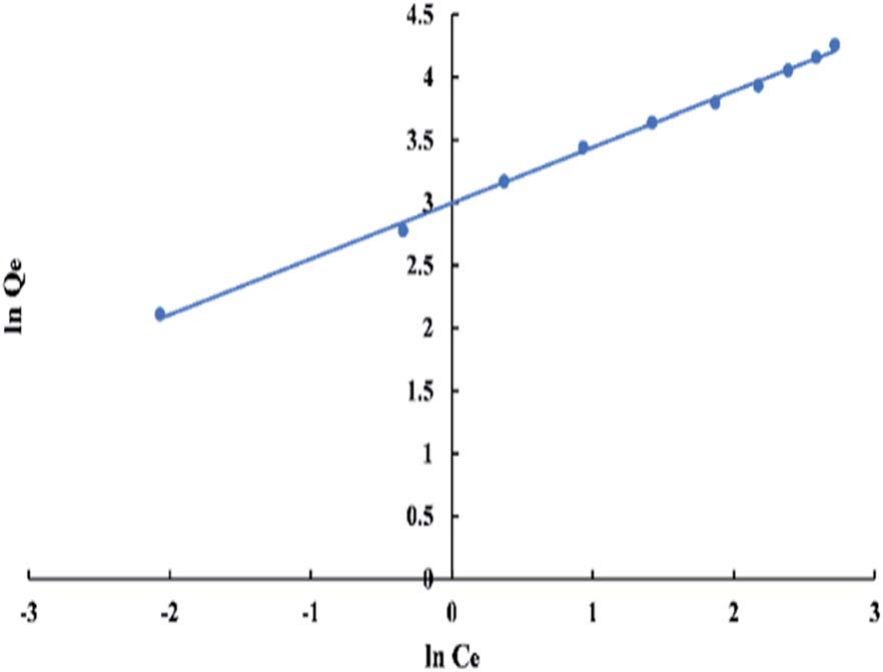
Freundlich isotherm for adsorption of BG onto ADPC at 298 K.

For Freundlich model, ln *Q*_e_*versus* ln *C*_e_ has a linear relationship as shown in “Fig. 9”, with an intercept equal to ln *K*_f_ and a slope equal to 1/*n*. The logarithmic form of Freundlich is given by the following equation:

5 ln *Q*_e_ = (1/*n*)ln *C*_e_ + ln *K*_f_

where “*K*_f_” is the Freundlich constant which represents the adsorption capacity of the adsorbent in (mg g^−1^) and “1/*n*”indicates the favourability of the adsorption process. If the value of “1/*n*” lay between zero and one it means adequate adsorption.^40^

**Fig. 10:**
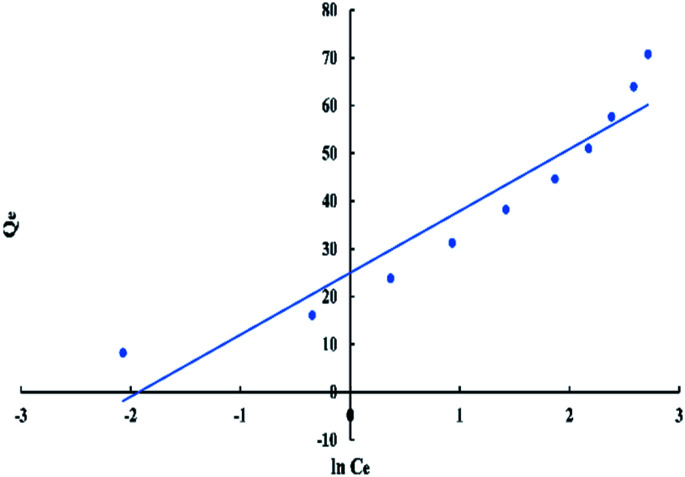
Temkin isotherm for adsorption of BG onto ADPC at 298 K.

Temkin model's equation can be expressed as:

6 *Q*_e_ = *q*_m_ln *K*_t_ + *q*_m_ln *C*_e_

where *K*_t_ and *q*_m_ are the Temkin constants related to adsorption capacity in (L mg^−1^) and heat of sorption in (J mol^−1^) respectively.^40^ The values of *q*_m_ and *K*_t_ can be obtained from the slope and intercept of the linear plot of *Q*_e_*versus* ln *C*_e_ as displayed in “Fig. 10”.

Dubinin–Radushkevich model can be represented by the following equations:

7 ln *Q*_e_ = ln *Q*_m_ − *Dε*^2^

**Fig. 11:**
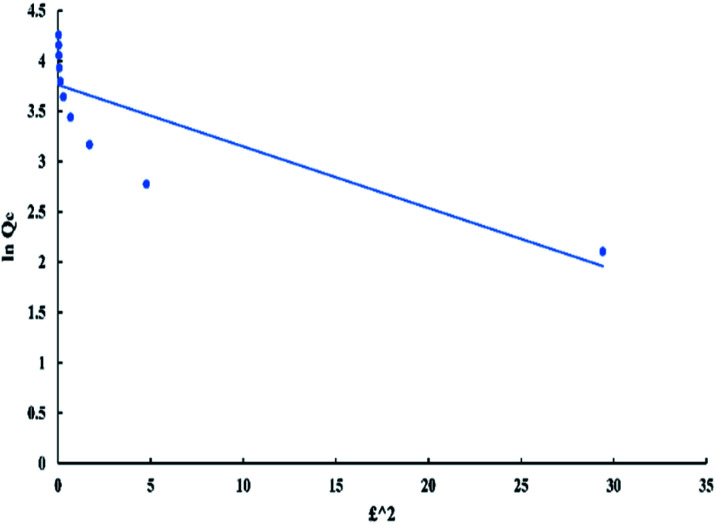
Dubinin isotherm for adsorption of BG onto ADPC at 298 K.

8 ε = *RT* ln ((*C*_e_ + 1)/*C*_e_)

where *ε* is Polanyi potential, *R* is gas constant (kJ mol^−1^ K^−1^), *T* is the absolute temperature in (K), and *D*, *Q*_m_ are Dubinin constants which related to the transfer of adsorption energy in (mol^2^ J^−2^) and the maximum adsorption capacity of the adsorbent in (mg g^−1^) respectively and they can be calculated from the slope and intercept of the plot between ln *Q*_e_ and *ε*^2^ in “Fig. 11”.^40^

### Kinetic study

Four kinetic models were used to fit the experimental data at 50 ppm initial dye concentration using a linear regression analysis method. The parameters of these models are summarized in “Table 4”.

**Table 4:** Kinetics study of BG dye onto activated date pits carbon (ADPC)

Kinetic models	Parameter value	Kinetic models	Parameter value
Pseudo-first-order model		Pseudo-second-order model	
*Q*_e_ “experimental” (mg g^−1^)	94.52828	*Q*_e_ “experimental” (mg g^−1^)	94.52828
*Q*_e_ “calculated” (mg g^−1^)	169.7719	*Q*_e_ “calculated” (mg g^−1^)	101.9671
*K*_1_ (min^−1^)	0.132227	K_2_ (g mg^−1^ min^−1^)	0.002101
*R* ^2^	0.7488	*R* ^2^	0.9986
Elovich model		Intra-particle diffusion model	
*α* (mg g^−1^ min^−1^)	152.2538	K_pi_ (mg g^−1^ min^−0.5^)	6.080824
*β* (g mg^−1^)	0.066326	C (mg g^−1^)	50.92069
*R* ^2^	0.9583	*R* ^2^	0.8833

**Fig. 12:**
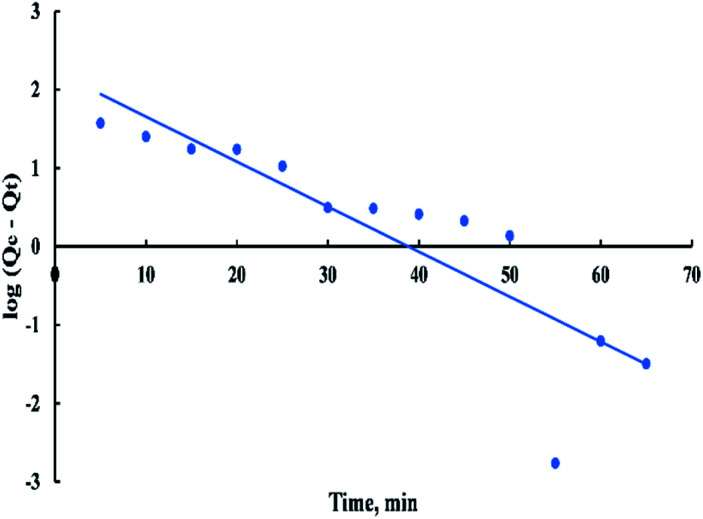
First order kinetic model for adsorption of BG onto ADPC at 298 K.

The pseudo-first-order rate expression is given as:

9 log(*Q*_e_ − *Q*_*t*_) = log(*Q*_e_) − ((*K*_1_^*t*^)/2.303)

where *Q*_*t*_ is the amount of dye adsorbed on the adsorbent at any time, *t* in (mg g^−1^), and *K*_1_ is the first-order rate constant. A “Fig. 12” of log(*Q*_e_ − *Q*_*t*_) *versus t* gives a linear relationship from which the value of *K*_1_ and *Q*_e_ can be determined from the slope and intercept.^54^

**Fig. 13:**
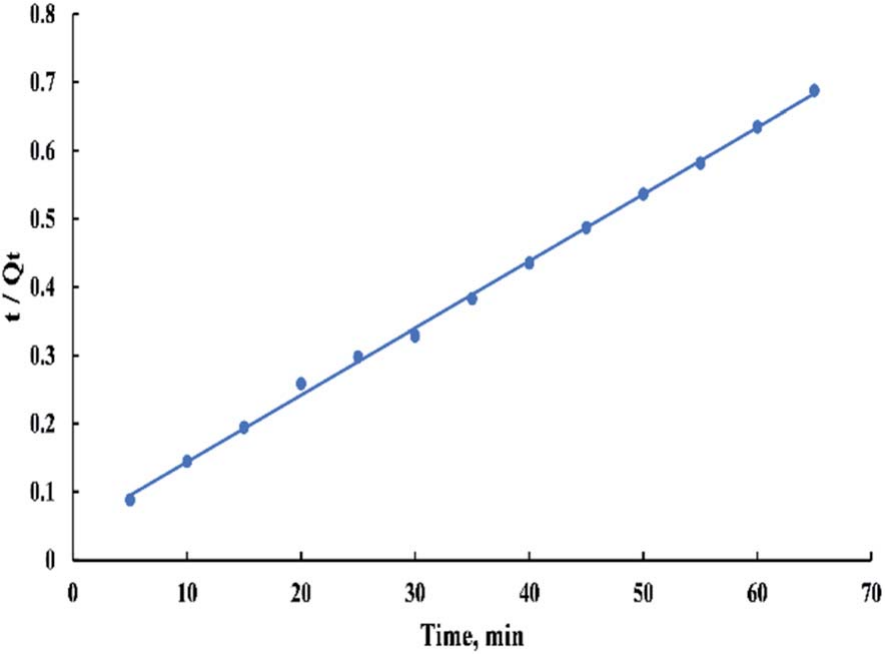
Second order kinetic model for adsorption of BG onto ADPC at 298 K.

The linearized form of the pseudo-second-order model is given as:

10 *t*/*Q*_*t*_ = (1/(*K*_2_*Q*_e_^2^)) + *t*/*Q*_e_

where *K*_2_ is the rate constant of the pseudo-second-order adsorption. The plot of *t*/*Q*_*t*_*versus t* in “Fig. 13” gives a linear relationship from which *Q*_e_ and *K*_2_ can be determined from the slope and intercept of the plot respectively.^55^

**Fig. 14:**
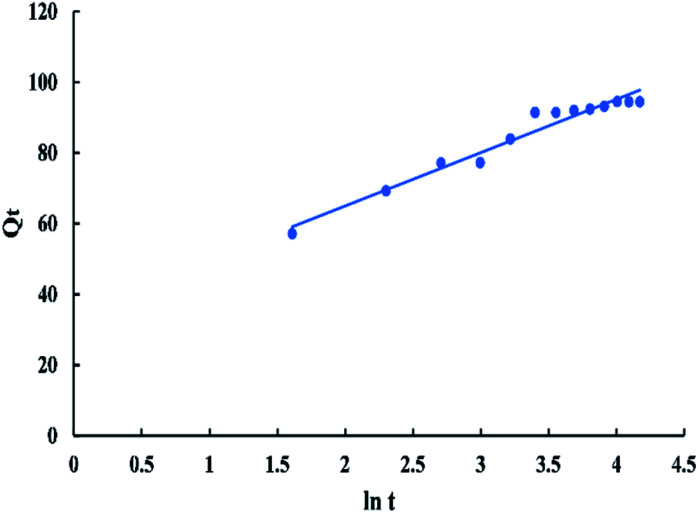
Elovich kinetic model for adsorption of BG onto ADPC at 298 K.

The Elovich model is generally expressed as:

11 *Q*_*t*_ = (1/(*β* ln[*αβ*])) + (1/(*β* ln[*t*]))

where *α* is the initial adsorption rate (mg g^−1^ min^−1^) and *β* is related to the extent of surface coverage and the activation energy for chemisorption (g mg^−1^).^55^ A plot of *Q*_*t*_*vs.* ln *t* in “Fig. 14” gives a linear relationship with a slope of “1/*β*” and an intercept of 1/*β* ln(*αβ*).

**Fig. 15:**
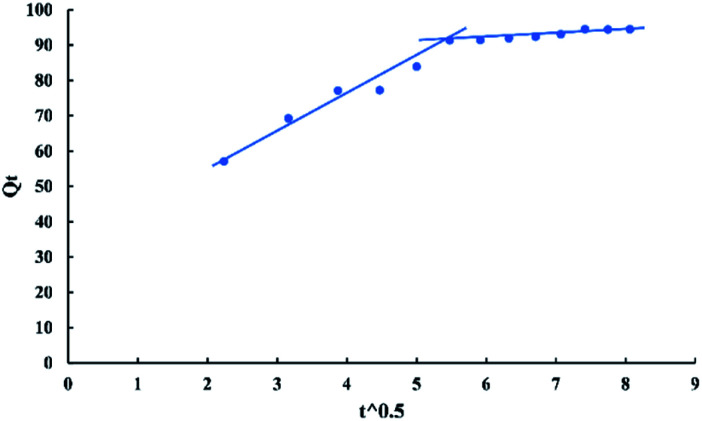
Intra-particle diffusion model for adsorption of BG onto ADPC at 298 K.

The intra-particle diffusion model used here refers to the theory proposed by Weber and Morris based on the following equation:

12 *Q*_*t*_ = *K*_pi_*t*^0.5^ + *C*

where *K*_pi_ (mg g^−1^ min^−0.5^) is the intra-particle diffusion rate constant, and *C* (mg g^−1^) describes the boundary layer thickness.^6^ The plot of *Q*_*t*_*versus t*^0.5^ in “Fig. 15” gives a linear relationship of slope (*K*_pi_) and an intercept (*C*).

### Thermodynamic study

The thermodynamic parameters that used to describe the adsorption process are the change of Gibbs free energy (Δ*G*, kJ mol^−1^), enthalpy (Δ*H*, kJ mol^−1^), and entropy (Δ*S*, kJ mol^−1^ K^−1^). They are calculated depending on the equilibrium experimental data obtained at different temperatures from 298.15 to 368.15 K and 50 ppm initial dye concentration by using the Van't Hoff equations as follow:

13 Δ*G* = −*RT* ln *K*

**Fig. 16:**
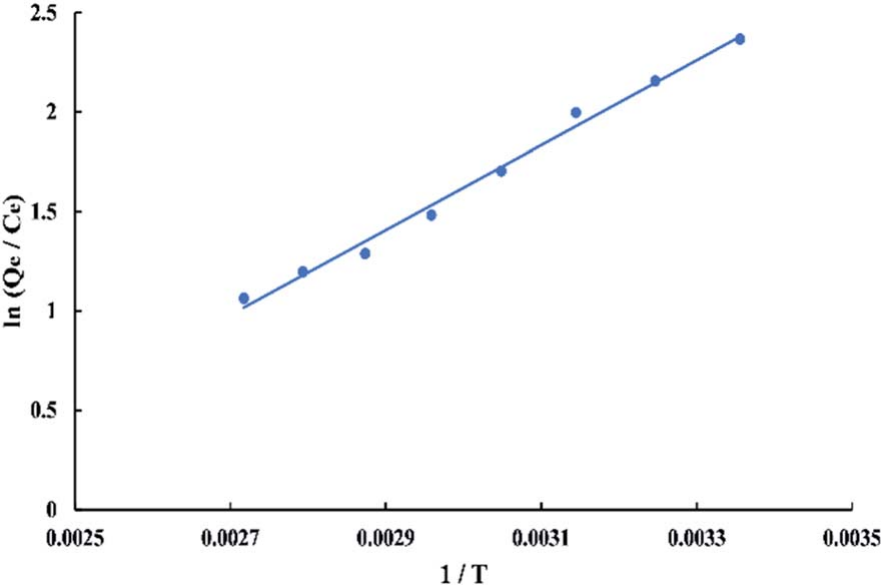
Graphical determination of Δ*H* and Δ*S* using Van't Hoff chart.

14 ln *K* = Δ*S*/*R* − (Δ*H*/*R*T)

where *R* is the ideal gas constant (8.314 J mol^−1^ K^−1^), *T* is the absolute temperature in kelvin, and *K* = *Q*_e_/*C*_e_. The enthalpy (Δ*H*) and entropy (Δ*S*) of the process were estimated from the slope and intercept of the plot of ln *K versus* 1/*T* as shown in “Fig. 16” which gives −17.7324 kJ mol^−1^ enthalpy and −0.039729 kJ mol^−1^ K^−1^ entropy at 298 K. Gibbs free energy (Δ*G*) was calculated using “eqn (13)” which gives negative values at all temperatures so, the adsorption of BG onto activated date pits carbon (ADPC) is physiosorptive because the free energy values fall in the range of (−20 to 0 kJ mol^−1^).^6^ Hence, the links between dye molecules and the adsorbent surface can be due to van der Waals or electrostatic attraction forces. The negative values of Δ*H* and Δ*S* indicate that the adsorption of BG onto ADPC is exothermic and spontaneous at low temperature.^40^

### Design of single-stage batch adsorber

**Fig. 17:**
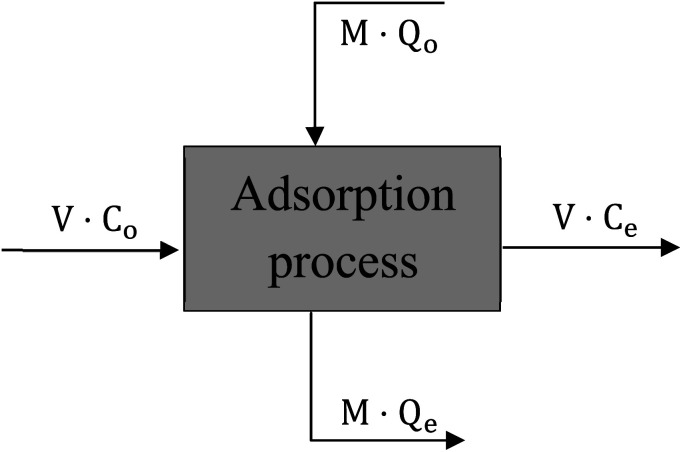
Single-stage batch adsorber unit.

The design of batch adsorber is considered the practicable step for using the equilibrium data resulted from experimental work by calculating the required adsorber volume and the optimum adsorbent weight.^40^ “Fig. 17” displays a schematic diagram for the single-stage batch adsorber where the initial concentration of the dye changes from *C*_o_ to *C*_e_ and the adsorption capacity of the adsorbent changes from *Q*_o_ to *Q*_e_.

The mass balance for the batch adsorber with a solution volume (*V*) and the amount of adsorbent (*M*) can be written as follow:

15 *MQ*_o_ + *VC*_o_ = *MQ*_e_ + *VC*_e_

16 *V*(*C*_o_ − *C*_e_) = *M*(*Q*_e_ − *Q*_o_)

By using the Freundlich adsorption isotherm model as it is the best-fitted model and by assuming that activated date pits carbon (ADPC) is a fresh adsorbent (*Q*_o_ = zero) so, “eqn (16)” can be rearranged as follow:

17 *M*/*V* = (*C*_o_ − *C*_e_)/*Q*_e_ = (*C*_o_ − *C*_e_)/[*K*_f_*C*_e_^1/*n*^]

**Fig. 18:**
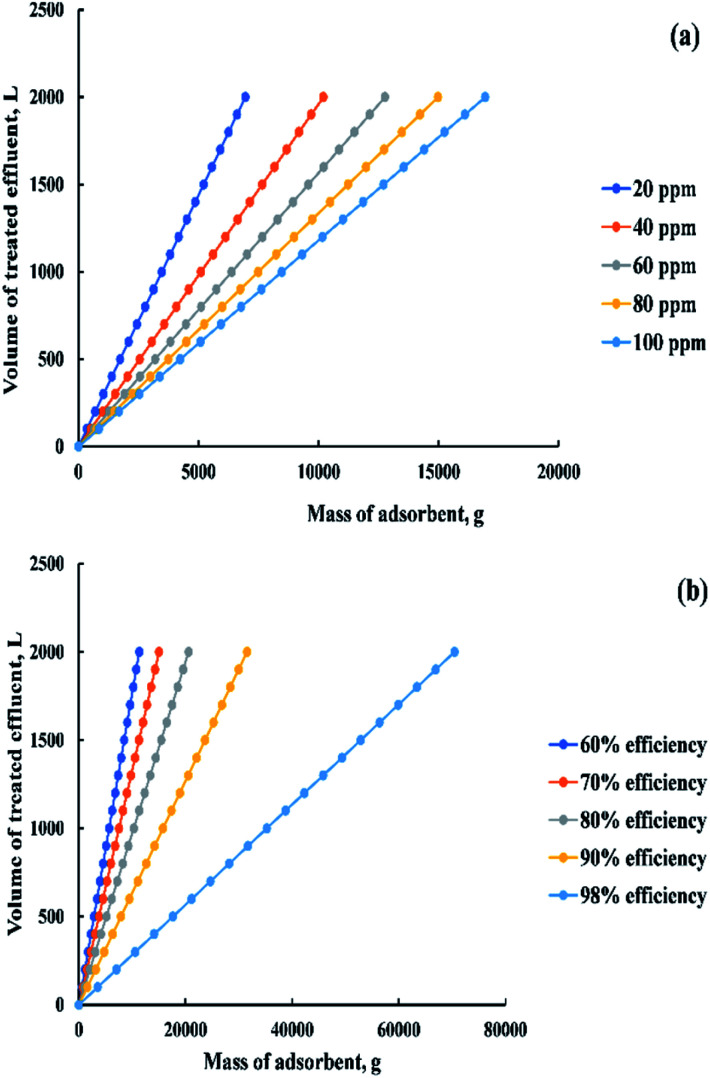
The relation between mass of the adsorbent and volume of the dye solution (a) at 85% efficiency and (20 : 100 ppm) initial dye concentration, (b) at 200 ppm initial dye concentration and efficiency from 60 to 98%.

Based on the previous equation, a relationship was drawn in “Fig. 18a” between the mass of adsorbent and the volume of dye solution that needs to be treated (100 : 2000 L) at a removal percent equal to 85% and a range of initial dye concentration from 20 to 100 ppm. Otherwise, a relationship was drawn in “Fig. 18b” between the previous variables but at different removal percent (60 : 98%) and constant initial dye concentration (200 ppm).

### Proposed mechanism for the process

The general methodology for any adsorption process can be summarized in the following stages:

(a) The dye molecules have to encounter the boundary layer effect for the adsorbent and diffuse from the boundary layer film onto the adsorbent surface.

(b) Diffusion of the dye molecules into the porous structure of the adsorbent where intra-particle diffusion of solute takes place in the adsorbed state or the liquid-filled pores of the particle.

According to the kinetic study, the pseudo-second-order is the best-fitted model for this process (*R*^2^ > 0.99) and from “Fig. 15” it's observed that the plot doesn't pass through the origin. Therefore, it can be concluded that the second stage is the rate-controlling stage.^6,40^

According to the thermodynamic study, it's clearly shown that the negative values of Δ*G* fall in the range of (−20 to 0 kJ mol^−1^) so, it can be concluded that the process is physiosorptive.^6^

Therefore, the process methodology can be deduced as follows:

(1) BG molecules encounter the boundary layer film and diffused rapidly onto the surface of activated date pits carbon (ADPC) due to the continuous shaking of bottles by the shaking water bath (Wisd laboratory instruments, DAHAN Scientific co., Ltd, 30, Korea).

(2) Intra-particle diffusion of BG molecules takes place in the porous structure of ADPC where hydrogen bonds (van der Waals or electrostatic attraction forces) were formed between BG molecules and the hydroxyl groups on the surface of ADPC until the process reaches the equilibrium state.

## Conclusion

This study showed that activated carbon prepared from date pits (ADPC) by chemical activation with phosphoric acid was a favorable adsorbent for the removal of brilliant green dye from aqueous solutions over a wide range of concentrations. The optimum experimental data were 55 min contact time, 0.06 g adsorbent dose, and basic medium pH = 8. Kinetic and Equilibrium studies were best fitted with the pseudo-second order kinetic model (*R*^2^ > 0.99) and Freundlich isotherm model (*R*^2^ > 0.99). The specific surface area of ADPC was 311.38 m^2^ g^−1^ which gives a convenient maximum monolayer adsorption capacity of 77.8 mg g^−1^. The negative values of Δ*G* were demonstrated that the process is physical adsorption and spontaneous at low temperatures concerning the negative values of Δ*H* and Δ*S* which proved that the reaction is exothermic and as the temperature increase, the solubility of dye molecules increase so removal percent decrease.

## Conflicts of interest

There are no conflicts to declare.

## Supplementary Material
